# A surface plasmon resonance immunoassay for the rapid analysis of methamphetamine in forensic oral fluid

**DOI:** 10.1002/jcla.22993

**Published:** 2019-08-02

**Authors:** Jiye Wang, Weixuan Yao, Fanwei Meng, Pengjuan Wang, Yuanzhao Wu, Binjie Wang

**Affiliations:** ^1^ Key Laboratory of Drug Prevention and Control Technology of Zhejiang Province, Department of Criminal Science and Technology Zhejiang Police College Hangzhou China; ^2^ Hangzhou Neoline Technology CO., LTD. Hangzhou China

**Keywords:** biosensor, forensic analysis, methamphetamine, oral fluid, surface plasmon resonance

## Abstract

**Background:**

Current chromatographic methods applied for the forensic analysis of methamphetamine are costly, time‐consuming, and require complicated pretreatment procedures. Thus, the rapid detection of methamphetamine is a critical and unmet need. In this study, a surface plasmon resonance (SPR) system based on indirect inhibitive immunoassay was designed for the analysis of methamphetamine in forensic oral fluid samples.

**Methods:**

For the inhibition immunoassay, the diluted oral fluid was mixed with methamphetamine antibody and then injected into the SPR sensor chip. The biosensor chip was constructed by covalently immobilizing of methamphetamine‐bovine serum albumin conjugate onto a carboxymethyl dextran surface at an optimized pH. The concentration of antibody was also optimized.

**Results:**

The SPR biosensor showed good sensitivity with a limit of detection of 0.44 ng/mL and was comparable or lower than the pre‐existing methods. The method was finally tested using oral fluid samples from 20 suspected drug abusers in forensic cases, and it provided an acceptable recovery of 113.2%, indicating good anti‐interference capability of the SPR sensor.

**Conclusion:**

The SPR biosensor was rapid, reproducible, and had a great potential approach for the forensic detection of methamphetamine.

## 1 INTRODUCTION

Methamphetamine (MA), a widely circulated “club drug,” has strong effects on the central nervous system, resulting in mental alertness and other symptoms.^1^ The abuse of MA continues to be one of the most concerning problems for public safety. The detection of drugs in biological specimens, typically urine, blood, and oral fluid, is important for the identification of drug addicts.^2^

In general, accurate detection methods in forensic laboratories mainly utilize either gas chromatography‐mass spectrometry (GC‐MS),^3^, ^4^, ^5^ high‐performance liquid chromatography (HPLC),^6^ or LC‐MS.^7^, ^8^, ^9^, ^10^, ^11^ These methods are sensitive and specific since they allow specific identification and accurate quantification of the target analyte. Due to the high complexity of biological samples and low concentrations of target analytes, these chromatographic methods usually require a series of sample pretreatment steps to eliminate matrices and enrich target compounds. Enzyme‐linked immunosorbent assay (ELISA), a quick and cheap approach, is the first line of screening utilized for the determination of the presence of an abused drug in biological samples. However, it cannot be used to reliably identify the tested drugs.^12^ Thus, there is a demand for a fast and direct method with minimal sample pretreatment for forensic drug analysis.

To this end, surface plasmon resonance (SPR), an optical sensing technique that investigates surface phenomena and generates a signal related to a change in refractive index at the metal was considered. It has the advantages of rapid‐response measurements with high sensitivity and specificity, without the need for intricate sample preparation. The value of SPR equipment is less than the currently used for MA analysis.

SPR has been known to be a capable analytical technique for the detection of various biological molecules, chemicals, and metal ion,^13^, ^14^, ^15^, ^16^, ^17^, ^18^, ^19^, ^20^, ^21^, ^22^, ^23^, ^24^ and as such, it is a promising tool for forensic drug analysis.^25^ There are limited reports related to the detection of MA using an SPR biosensor. Sakaia et al 26 developed an SPR sensor based on the immunoreaction between methamphetamine‐bovine serum albumin (MA‐BSA) and the antibody, and validated its feasibility for the sensitive response for MA. Cao et al 27 proved its usefulness for quantitative analysis of MA in spiked human serum.

Oral fluid testing for MA has emerged as a promising alternative to urine and blood testing. Oral liquid can be easily and noninvasively collected under observation, thereby eliminating the problem of substitution or adulteration that are often associated with urine collection. Furthermore, previous studies have found that the MA concentration in oral liquid could correlate well with those in blood and plasma.^28^, ^29^

In this study, an SPR sensor based on indirect competitive immunoassay enabling the rapid detection of MA is presented. The immobilization process and MA‐antibody concentration were individually optimized. The specificity of the sensor was evaluated by testing the oral fluid samples from forensic cases. High sensitivity and satisfactory recovery were obtained. This work sheds some light on developing SPR sensor as a powerful technique for the forensic monitoring of MA.

## 2 EXPERIMENTAL

### 2.1 Chemicals and reagents

Bovine serum albumin (BSA), MA‐BSA conjugate, and anti‐MA monoclonal antibody (MA‐Ab) were purchased from Hangzhou Clongene Biotech Co., Ltd. The amine‐coupling reagents containing N‐hydroxysuccinimide (NHS, 98%) and 1‐ethyl‐3‐[3‐dimethylaminopropyl] carbodiimide hydrochloride (EDC, ≥99.0) were obtained from Sigma‐Aldrich. Phosphate buffered saline (PBS) and ethanolamine were purchased Sigma‐Aldrich. Methamphetamine (1 mg/mL in methanol) was provided by the Public Security Bureau of Hangzhou. Acetic acid‐sodium salt was purchased from Sinopharm Chemical Reagent Co., Ltd. Acetic acid was purchased from Shanghai Lingfeng Chemical Reagent Co., Ltd.

### 2.2 Instrumentation

An automatic flow injection system (BT100F‐1) was used for sample injection. SPR measurements were performed on a Biacore 3000 system (Swit). The MA diagnostic kit (colloidal gold) was purchased from ACON Biotech Co., Ltd. The CM5 sensor chip (20 mm × 10 mm × 0.5 m) was obtained from the General Electric Company. A ThermoFisher Micro 21 centrifuge and an Amicon ultra‐0.5 centrifugal filter unit (Merck KGaA) were used for the purification of samples.

### 2.3 Immobilization procedure of the MA‐BSA conjugate

The immobilization of MA‐BSA conjugate consisted of the following three steps. First, the chip was mounted on the SPR platform. A mixture (1:1, v/v) of 100 mmol/L NHS and 400 Mmol/L EDC was injected over the chip to activate the surface carboxyl groups. Second, MA‐BSA conjugate (1 mg/mL) in 10 mmol/L acetic acid‐sodium acetate buffer was flowed through the activated chip. Third, the unused carboxylic terminals were blocked by injecting 100 µL of 1 mol/L ethanolamine (pH 8.5). These procedures were done at a flow rate of 20 µL/min.

### 2.4 SPR measurement

An indirect competitive immunoassay format was used for the detection of MA. The MA‐BSA conjugate was immobilized and used as the sensing bioactive surface. In measurement, 40 µL samples containing a fixed antibody concentration were incubated with solutions of varying MA concentrations at a ratio of 1:1 (v/v), and this mixture was flowed over the antigen‐coated chip at a flow rate of 20 µL/min at room temperature, which took about 3 minutes. Since antibody binding to the immobilized conjugate was inhibited by the presence of the analyte, lower analyte concentrations resulted in high SPR signals and vice versa. Finally, 10 µL of 50 mmol/L NaOH solution was injected to regenerate the sensing surface, and the regeneration process took approximately 2.5 minutes.

### 2.5 Calibration curve

The stock solution of MA (1 mg/mL) was diluted with PBS, and then mixed with an equal volume of 25 µg/mL MA‐Ab, to obtain final concentrations of 0.06, 0.12, 0.24, 0.49, 0.98, 1.95, 3.91, 7.81, 15.63, 31.25, 62.5, and 125 ng/mL, respectively. Then, 40 µL of the solution was passed over the sensor chip at a flow rate of 20 µL/min. A standard calibration curve was obtained by plotting SPR signal versus MA concentration.

### 2.6 Oral fluid collection

The specificity of the sensor was evaluated by testing frozen oral fluid samples collected from 20 suspected drug abusers in forensic cases. All samples were collected in accordance with the ethical guidelines and permission of the institutional review board (IRB). For analysis, the frozen oral fluid was stable at room temperature for ten minutes. Then it was added to an Amicon ultra‐0.5 centrifugal filter and centrifuged at 12 000 rpm for 10 minutes. The supernatant was mixed with Ab solution and injected into the SPR system for analysis.

## 3 RESULTS AND DISCUSSION

### 3.1 SPR immunoassay format

**Figure 1:**
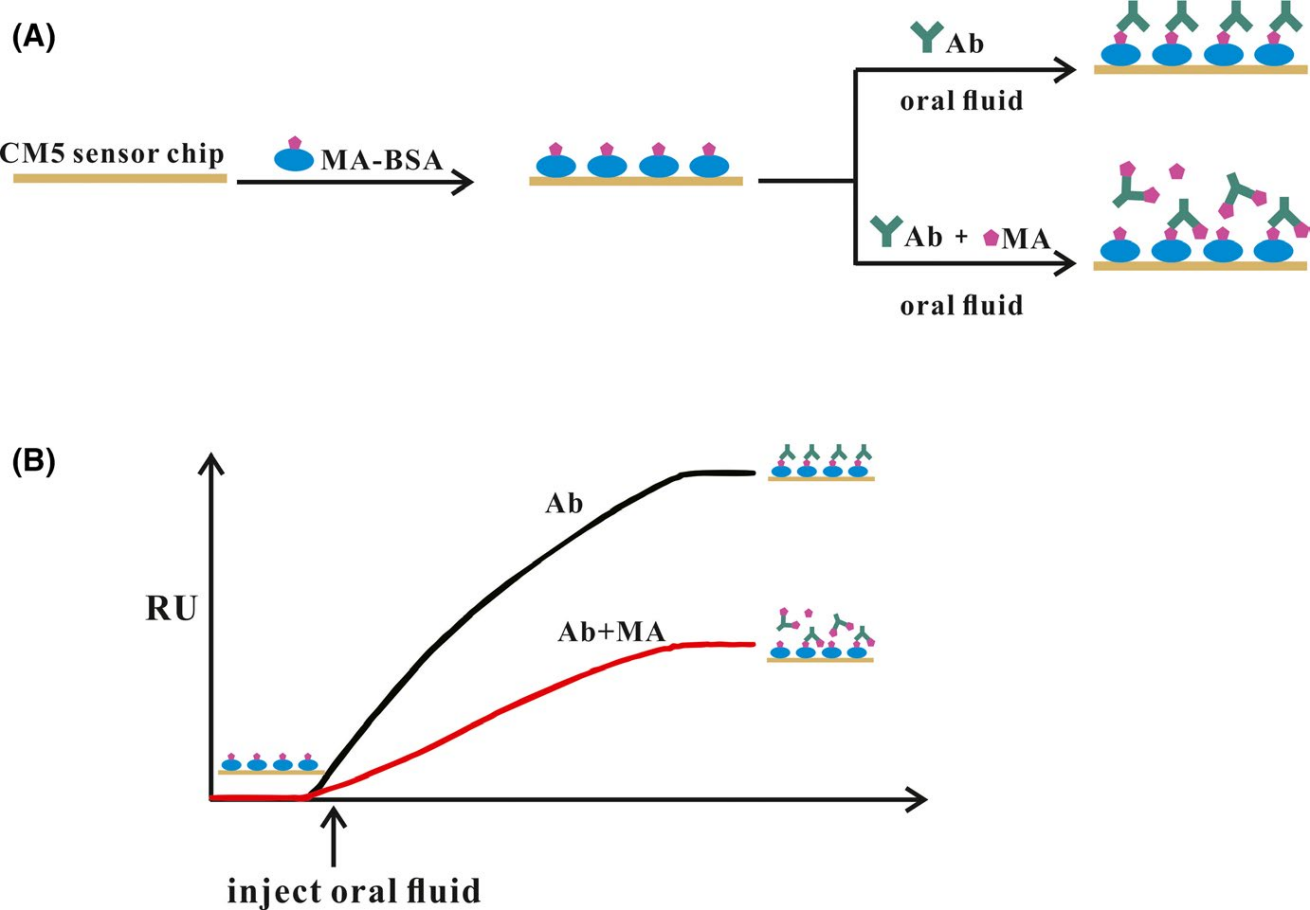
Scheme of the SPR immunoassay for MA

The molecular weight of the analyte is a key factor when selecting the immunoassay format. MA (MW: 149.237 g/mol) itself has no immunogenicity, thus an inhibitive assay to indirectly measure the concentration of MA was adopted. Alternatively, in an indirect format, the immobilization of target molecules with a conjugated protein onto the sensing chip provides a higher degree of robustness and reusability even under acid and basic conditions^26^. In this approach, the conjugate antigen (MA‐BSA) was firstly immobilized on a sensor chip and was then exposed to PBS solutions containing MA and antibody to allow the immunoreaction to take place. As shown in Figure 1, MA functions as an inhibitor to the immunoreaction between the antibody and MA‐BSA, and a strong decrease in response occurred with the addition of MA, suggesting the presence of binding affinity between MA‐BSA and MA‐Ab.

### 3.2 Optimization of immobilization of MA‐BSA conjugate

**Figure 2:**
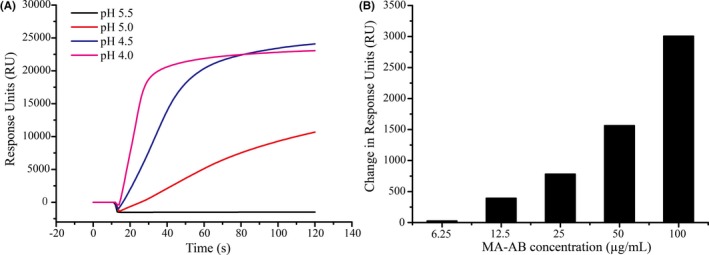
Optimization of immobilization of BA‐BSA conjugate on sensor chip at varying pH (A), and dependence of SPR signal against MA‐antibody concentration (B)

MA‐BSA was covalently immobilized to the carboxymethyl dextran matrix on the sensor chip through EDC/NHS esters under acidic condition (acetic acid‐sodium acetate buffer). To achieve the best sensor performance, MA‐BSA conjugate (1 mg/mL) was immobilized at different pH levels: 4.0, 4.5, 5.0, and 5.5. The correlation between resonance signals and the pH value is shown in Figure 2A. It can be seen that the decreased pH value led to a gradually rising resonant signal until approaching saturation at pH 4.0‐4.5. A pH of 5.0 is suitable for better assay sensitivity and discernible signals for a wider range of analyte concentrations. Thus, in order to favor competition, we immobilized MA‐BSA conjugate at pH 5.0 and tested several MA‐Ab concentrations in the following experiments.

### 3.3 Optimization of MA‐Ab concentration

The MA‐BSA immobilized chip was exposed to the flow of several MA‐Ab concentrations including 6.25, 12.5, 25, 50, and 100 µg/mL, respectively (Figure 2B). The resonance signals increased with increasing concentrations of the antibody, and there was no indication of surface saturation within the tested concentration range. However, at lower concentration levels of 6.25 and 12.5 µg/mL, the responding resonance signals were relatively low, which might lead to a relatively narrow linear range. Conversely, using higher antibody concentrations of 50 and 100 µg/mL would cause an increase in the cost. Therefore, an MA‐Ab concentration of 25 µg/mL was deemed appropriate for the MA sensing assay.

### 3.4 Reusability of the sensor chip

**Figure 3:**
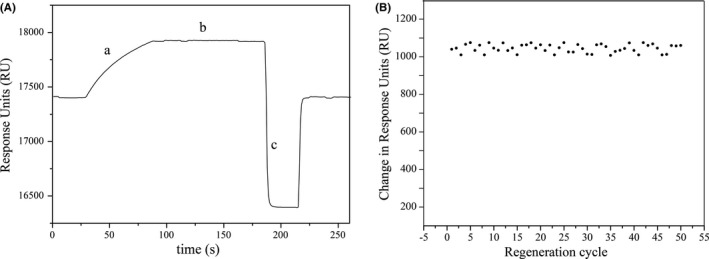
Chip regeneration process with 50 mmol/L NaOH (A). (a) Start of sample injection, (b) stabilized signal, and (c) start of regeneration. Reusability of the MA sensor chip during 50 successive binding‐regeneration cycles (B)

In order to assess the reusability and robustness of the sensing chip and the reproducibility of the measurements, we tested the chip reaction process using 50 mmol/L NaOH as a regeneration reagent. As seen in Figure 3A, the injection of a mixture of MA and MA‐Ab caused an initial increase in the SPR signal (indicated as position a) and its subsequent stabilization (position b). The regeneration was then applied (position c), after which the basal line was finally recovered. Correspondingly, the resonance signal of the initial baseline was 1748 RU, and it returned to 1951 RU upon injection of 10 µL of 50 mmol/L NaOH, with a minute variation of 0.17%, indicating the complete removal of bound antibody from the sensor surface.

The recycling experiments in Figure 3B showed that the same chip could be reused at least 50 times in this way without a significant decrease in response and with a coefficient of variation (CV) of 2.15%. These results confirmed the reliability and robustness of the immobilized chips.

### 3.5 Sensitivity and limit of detection of MA by the SPR biosensor

**Figure 4:**
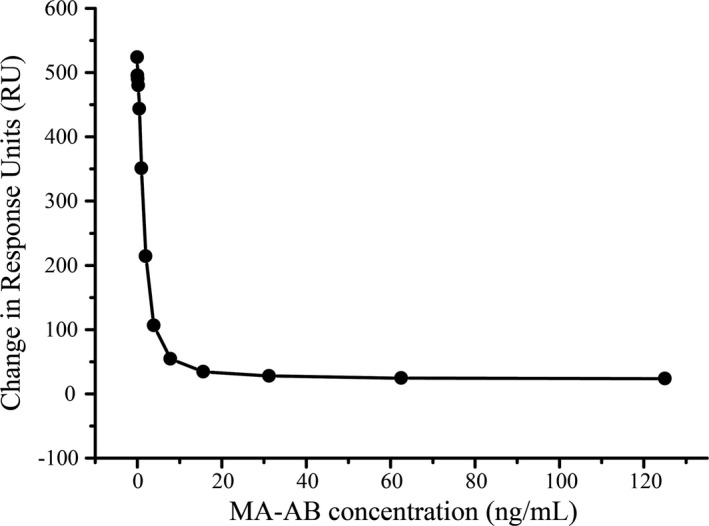
Dependence of SPR signals on the MA concentration

The SPR response signal of each solution containing a fixed concentration of MA‐Ab (25 µg/mL) and varying concentrations of MA (0‐125 ng/mL) are plotted in Figure 4. Clearly, SPR signals were sensitive to the change in MA concentrations from 0.06‐15.63 ng/mL. With an increase up to 15.63 ng/mL, SPR signals decreased fairly steeply due to the inhibition effect of MA. The data points fit well with the four‐parameter logistic model,^18^ with a correlation coefficient (*r* ^2^) of 0.9991. Above 15.63 ng/mL, the signals were insensitive to the MA concentrations, because the whole antibody was inactivated by the excessive amount of MA. It was notable that the present sensor could detect MA at concentrations less than 15.63 ng/mL. The limit of detection (LOD), determined as 3 × noise inhibition of the maximum SPR signal,^27^ was estimated to be 0.44 ng/mL.

### 3.6 Comparison with other reported methods

A comparison among different detection techniques for MA in biological samples is shown in Table 1. Conventional chromatographic techniques, including GC‐MS, HPLC, and LC‐MS, require extraction procedures prior to analysis.^3^, ^4^, ^5^, ^6^, ^7^, ^8^, ^9^, ^10^, ^11^ This SPR immunoassay is suitable for the direct analysis of oral fluid samples without any pretreatment steps. It requires only sub‐microliter levels of sample and avoids the use of organic solvents. In general, this SPR method has the advantages of simplicity, allowing the fast and real‐time determination of MA, while achieving comparable or ever lower detection limits than pre‐existing methods.

**Table 1:** Comparison of several methods used for the detection of MA in biological specimens

Method	Limit of detection	Ref.
Magnetic solid‐phase extraction coupled with GC‐MS	0.044 ng/mL	3
Liquid‐liquid extraction coupled with GC‐MS	5 ng/mL	4
Liquid‐phased microextraction coupled with HPLC	0.01 µg/mL	6
Supramolecular solvents coupled with LC‐MS/MS	5 ng/mL	9
Supported liquid extraction coupled with LC/MS	5 ng/mL	11
SPR biosensor	0.44 ng/mL	this work

### 3.7 Determination of MA in oral fluid samples

**Figure 5:**
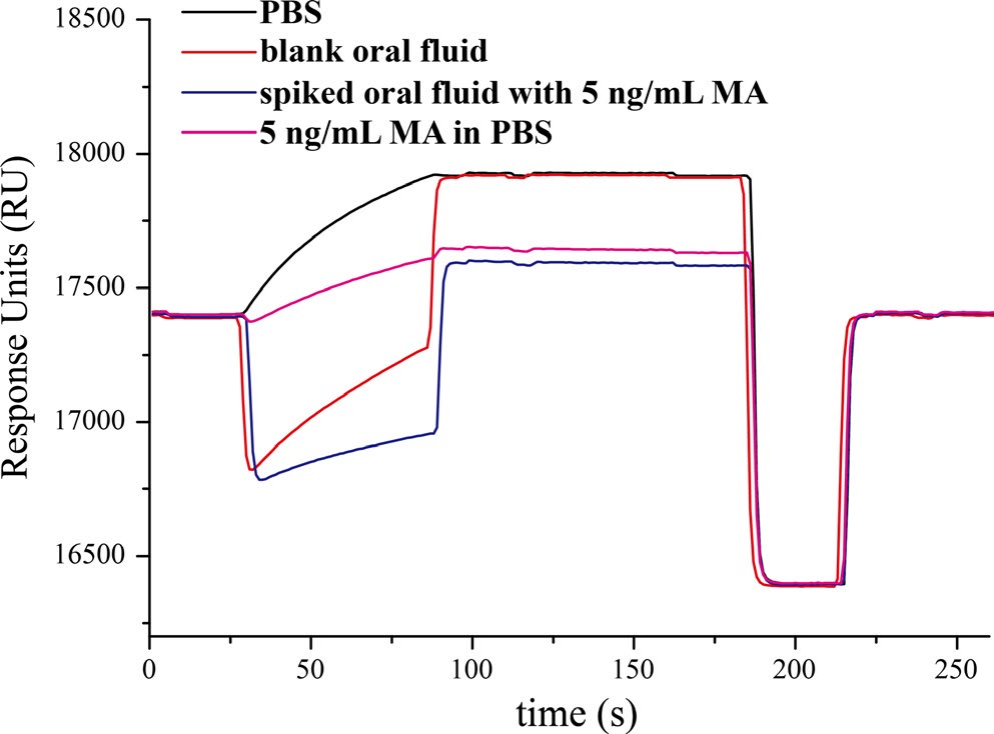
The SPR curves of standard samples and oral fluid samples

According to the obtained assay features, this SPR‐based approach can be useful for the determination of MA in oral fluid samples from 20 suspected drug abusers in forensic cases. The results are listed in Table 2. MA could be found in 17 MA abusers' oral fluid samples, thus confirming investigators' suspicions of drug exposure. Recoveries of spiked oral fluid sample at spiked level (5 ng/mL) were conducted to study the matrix effects. The average recovery was found to be 113.2%, with RSD of 3.1%. Besides, the real‐time SPR curves of blank oral fluid and spiked oral fluid are shown in Figure 5. The results demonstrated a good anti‐interference capability and application feasibility of the SPR biosensor in quick confirmation and quantification of MA abuse.

**Table 2:** Analytical results of oral liquid samples from forensic cases by SPR biosensor and colloidal gold methods

Oral liquid	MA diagnostic kit (colloidal gold)	SPR biosensor	
		Detected (ng/mL)	RSD (%)
1	+^a^	>15.63	7.32
2	+	>15.63	6.81
3	+	>15.63	8.46
4	+	>15.63	9.82
5	+	>15.63	3.98
6	+	>15.63	1.36
7	−b	10.41	6.91
8	−	7.51	3.65
9	−	10.54	7.53
10	−	4.37	7.37
11	−	8.31	6.53
12	−	5.44	8.10
13	−	12.45	5.46
14	−	6.24	7.20
15	−	3.52	4.80
16	−	0.96	2.97
17	−	0.56	3.85
18	−	nd^c^	3.54
19	−	nd	8.95
20	−	nd	4.17

a MA‐positive.

b MA‐negative.

c not detected.

In forensic toxicology, MA diagnostic kits are a rapid visual gold colloidal method that is often used for the determination of the presence of an abused drug in biological specimens, at an LOD value of approximately 1000 ng/mL. Six saliva samples were founded to be positive. Taken together, the SPR biosensor provides a relatively accurate method for the MA detection in biological specimens.

## 4 CONCLUSION

This work highlights a sensitive and reusable SPR immunosensor for the simple and rapid forensic monitoring of MA in oral fluid samples. It is worth mentioning that this sensor allows direct injection of oral fluid after centrifugation and greatly reduced the analysis time to 3 minutes, while showing a comparable or lower LOD value (0.44 ng/mL) to the current methods that are sensitive enough for confirmation of MA abuse. Further validation and implementation was  evaluated using human oral fluid samples in forensic cases. This is the first time to realize such a sensitive detection of MA in forensic samples using an SPR‐based sensor. This method is simple, rapid, sensitive, and reproducible, and more importantly, it paves the way for the potential application of SPR biosensors in the forensic laboratory analysis of illicit drugs.
